# Cytomegalovirus infection after renal transplantation

**DOI:** 10.25122/jml-2021-0209

**Published:** 2022-01

**Authors:** Mohammed Younus Naji Al Atbee, Hala Sami Tuama

**Affiliations:** 1. Department of Nephrology, College of Medicine, University of Basrah, Basrah, Iraq; 2. Beradaiya Primary Health Care, Basrah, Iraq

**Keywords:** Cytomegalovirus (CMV), renal transplantation, alanine aminotransferase (ALT), serum creatinine

## Abstract

Renal transplant patients show a high prevalence of cytomegalovirus (CMV) infection after the procedure. This study was conducted to assess the prevalence and factors associated with the incidence of CMV infection among renal transplant patients. A total of 100 patients were recruited in this study. The CMV load in the blood of each patient was assessed using the technique of polymerase chain reaction (PCR). The serostatus of all recipients and donors was examined preoperatively and those of the recipients again postoperatively. The association of CMV load was assessed with the following factors: age, gender, alanine aminotransferase (ALT) and serum creatinine levels, types of immunosuppressive and induction regimens, preoperative diabetes status, and serological virologic response (SVR) at 12 weeks postoperatively. Our findings showed that CMV incidence was significantly higher in middle-aged patients (62 of 66 patients, 93.9%; p=0.0001). Furthermore, about 88.2% of patients induced by anti-thymocyte globulin (ATG) showed a high viral load, significantly higher than the proportion of CMV-positive patients induced by basiliximab (p=0.001). In addition, a higher proportion of CMV-negative recipients who received the graft from CMV-positive donors and vice-versa were CMV-positive postoperatively. Administration of Valcyte 450 showed 100% efficiency in decreasing the CMV load in the patients. Among all the assessed factors, only the age of the recipients, type of induction therapy used, and the preoperative serostatus of both donors and recipients were significantly associated with the postoperative CMV incidence among the patients.

## Introduction

Cytomegalovirus (CMV) is a double-stranded (ds) DNA virus that belongs to the Herpesviridae family and infects humans. Around 50% of the global human population suffers from CMV infection. The incidence of CMV infection is also extremely high among solid organ transplant recipients. It is considered a major risk factor responsible for the deterioration of kidney graft function and graft failure, mainly responsible for high morbidity and mortality rates post-renal transplant. The prevalence of CMV infection among renal transplant recipients is primarily attributed to the drug-induced immunosuppression post-transplantation procedure. Such immunosuppression mainly targets the recipient’s T cells, which often leads to the activation and replication of CMV. With a diameter ranging from 150–200 nm, CMV is the largest known human herpesvirus [1]. Previous studies have shown a CMV seroprevalence of 70–90% among the adult human population [2, 3]. In the general population, the first CMV infection usually occurs at a young age, after which the subsequent infections have been reported to be recognized by CD34+ myeloid progenitors, megakaryocytes, dendritic cells, and CD14+ monocytes. However, in cases of immunosuppression, such as AIDS and renal transplantation, CMV infection often gets reactivated [4, 5]. The viral reactivation is mediated by the reduction in the activity of CD8+ cells and the induction of cytokines activity, such as tumor necrosis factor-alpha (TNF-α) and interleukin-1β (IL-1β). Clinically, CMV infection is manifested as a viral invasion of various tissues and organs, such as the liver, gastrointestinal tract, renal graft, bone marrow, lungs, and retina. CMV infection has been deemed a major cause of graft rejection in post-renal transplant recipients. Sadegal *et al.* showed that CMV infection causes a 1.6-fold increase in the risk of acute renal graft rejection [6]. One of the most crucial factors that affect the risk of CMV infection in post-renal transplant recipients is the preoperative infection status of both donors (D) and recipients (R). A combination of D+/R- (donor positive and recipient negative for infection) leads to the highest risk of infection among the post-transplant recipients.

It is noteworthy that CMV infection is clinically manifested in two ways, as a disease or an infection. CMV disease is characterized by different symptoms, including asthenia, fever, leukopenia, myalgia, hepatic enzyme alterations, or thrombocytopenia. In the case of CMV disease, the virus might also invade various tissues or organs, such as the liver, kidneys, bone marrow, gastrointestinal tract, lungs, and retina. On the other hand, in cases characterized only by CMV infection, symptoms are absent; however, viral activation and replication are still detectable [1]. The effects of CMV infection could be both direct (infection and disease) as well as indirect (secondary infections by other pathogens, graft dysfunction, graft rejection etc) [4].

Previous studies have reported PCR to be one of the most sensitive and recommended diagnostic methods of CMV infection. This method is based on the assessment of viral load. A positive viral load is considered an independent indicator of CMV infection. The only disadvantages of this technique are that it is time-consuming, there is no standardized PCR protocol for CMV detection, and it varies with different laboratories. In this study, we investigated the prevalence of CMV infection and its predictive factors among post-renal transplant patients.

## Material and Methods

### Patient recruitment

We recruited 100 individuals who underwent renal transplantation in the Nephrology Department of the Basrah Teaching Hospital, Basrah, Iraq. The demographic characteristics of all the patients were recorded and reviewed for the presence of CMV one-month post-transplant in each patient. None of the patients received CMV prophylaxis prior to the study. During the study, all patients underwent PCR every month to assess the CMV viral load. Prior to the renal transplantation procedure, the serostatus of all the recipients (R) and their respective donors (D) was recorded. Based on their serostatus, the patients were divided into four groups: when both R and D were CMV-negative (R negative D negative) when both R and D were CMV-positive (R positive D positive), when only R was CMV-positive (R positive D negative), and when only D was CMV-negative (R negative D positive). Furthermore, the alanine aminotransferase (ALT), urea, creatinine, and blood sugar levels were also recorded for all patients. Prior to the analyses, informed consent was obtained from all the patients.

### Polymerase chain reaction (PCR)

CMV quantification in the blood samples was done using PCR. The blood samples of all the patients were collected in EDTA tubes. The samples were then subjected to CMV real-time PCR (RT-PCR) conducted according to the COBAS® AmpiPrep CMV Test (Roche Molecular Diagnostics) as per the manufacturer’s instructions. The PCR results were expressed as copies/mL. The detection range of this technique is 150–10000000 copies/mL. One copy of CMV DNA viral load is equivalent to 0.91 International Units (IU) according to the WHO standard guidelines for human CMV techniques for nucleic acid amplification. The CMV DNA for a patient to be defined as CMV is significantly high in this study: >800 copies.

### Drug administration

As per the standard guidelines, the patients received either a tacrolimus-based regimen or cyclosporine-based regimen for immunosuppression and were induced by either basiliximab or antithymocyte globulin (ATG) to prevent the rejection of the kidney graft. Furthermore, postoperatively, in the case of CMV-positive patients, Valcyte 450 tablet was administered twice daily based on the glomerular filtration rate (eGFR) and serum creatinine levels estimated for 12 weeks. Response to oral therapy was assessed via a follow-up examination of the HCV viral load after 12 weeks. Sustained virologic response (SVR) was defined as negative HCV viral load (aviremia) at 12 weeks post-antiviral therapy.

### Statistical analysis

All the statistical analyses were conducted using the Statistical Package for Social Sciences (SPSS) 21. The statistical significance of the differences among the various variables was assessed using Pearson’s Chi-Square test. A p-value of less than 0.05 was considered statistically significant.

## Results

### Patient characteristics and CMV load

In this cross-sectional study, a total of 100 patients were recruited. Of the 100 patients, 74 (74%) and 26 (26%) patients were male and female, respectively. The male: female ratio was 2.8:1. The ages of all the patients ranged from 6–65 years, with a mean age of 39±6.5 years. Overall, the mean CMV viral load ranged from 450–2000000 copies/mL with a mean viral load of 1790058±590918.13 copies/mL (Table 1). Overall, the samples of 19 patients (19%) contained <800 copies CMV DNA/mL, whereas the samples of 81 patients (81%) contained >800 copies CMV DNA/mL.

**Table 1.** Mean age and Cytomegalovirus (CMV) viral load.

			**CMV viral load**		**Total**
			**<800 copies**	**>800 copies**	
**Age**	**10–30 years**	**Count**	11	16	27
		**%**	40.7%	59.3%	
	**31–50 years**	**Count**	4	62	66
		**%**	6.1%	93.9%	
	**50–60 years**	**Count**	4	3	7
		**%**	57.1%	42.9%	
Total		**Count**	19	81	100
		**% of Total**	19.0%	81.0%	100.0%

CMV – Cytomegalovirus.

### CMV viral load based on age and gender

We divided the patients into three groups based on their age, viz. <30 years, 31–50 years, and >50 years (Table 2). 27, 66, and seven patients belonged to the age groups of 30 years, 31–50 years, >50 years, respectively. In the above mentioned three groups, 16 (59.3%), 62 (93.9%), and three (42.9%) patients were CMV-positive, respectively. The statistical analyses revealed a significantly higher CMV incidence in middle-aged recipients (X^2^=22.09, p=0.0001). As mentioned in the previous subsection, out of the 100 recruited patients, 74 (74%) and 26 (26%) patients were male and female, respectively (Table 3). Around 78.4% and 21.6% male patients and 88.5% and 11.5% female patients were CMV-positive and CMV-negative, respectively. However, the viral load among the patients did not differ significantly based on gender (X^2^=1.271, p=0.26).

**Table 2.** Distribution of CMV viral loads according to age.

	**Age**	**CMV viral load**
**Mean**	39.7000	1790058.0000
**Total number**	100	100
**Standard Deviation**	6.51261	590918.13119
**Minimum**	6.00	450.00
**Maximum**	65.00	2000000.00

CMV – Cytomegalovirus.

**Table 3.** Distribution of CMV viral loads according to gender.

			**CMV viral load**		**Total**
			**<800 copies**	**>800 copies**	
Gender	**Female**	**Count**	3	23	26
		**%**	11.5%	88.5%	
	**Male**	**Count**	16	58	74
		**%**	21.6%	78.4%	
Total		**Count**	19	81	100
		**% of Total**	19.0%	81.0%	100.0%

CMV – Cytomegalovirus.

### CMV viral load based on ALT levels

Among the recruited patients, the ALT levels of 86 and 14 patients were high and normal, respectively (Table 4). Sixty-eight (79.1%) patients with high and 13 (92.9%) patients with normal ALT levels were CMV-positive. However, the CMV load was not significantly different between the patients with normal and high ALT levels (X^2^=1.487, p=0.223).

**Table 4.** Distribution of CMV viral loads according to ALT levels.

			**CMV viral load**		**Total**
			**<800 copies**	**>800 copies**	
ALT	**Normal ALT**	**Count**	1	13	14
		**%**	7.1%	92.9%	
	**High ALT**	**Count**	18	68	86
		**%**	20.9%	79.1%	
**Total**		**Count**	19	81	100
		**% of Total**	19.0%	81.0%	100.0%

CMV – Cytomegalovirus; ALT – Alanine Aminotransferase; Pearson’s Chi-Square = 1.487, p-value = 0.223.

### CMV viral load based on immunosuppressive and induction regimens

Twenty-four and 76 patients were induced by basiliximab and ATG, respectively, to reduce the probability of graft rejection. Among the basiliximab- and ATG-induced patients, 14 (58.3%) and 10 (41.7%) patients and 67 (88.2%) and 9 (11.8%) patients were CMV-positive and -negative, respectively. The difference in the CMV viral loads was statistically significant (p=0.001) (Table 5).

**Table 5.** Distribution of CMV viral loads according to the induction therapy.

			**CMV viral load**		**Total**
			**<800 copies**	**>800 copies**	
Induction therapy	**Basiliximab induction**	**Count**	10	14	24
		**%**	41.7%	58.3%	
	**ATG induction**	**Count**	9	67	76
		**%**	11.8%	88.2%	
Total		**Count**	19	81	100
		**% of Total**	19.0%	81.0%	100.0%

CMV – Cytomegalovirus; ATG – Anti-Thymocyte Globulin; Pearson’s Chi-Square = 10.542, p-value = 0.001.

Furthermore, 35 and 65 patients were treated with tacrolimus- and cyclosporine-based immunosuppressive regimens. Among the tacrolimus- and cyclosporine-based regimen-treated patients, 31 (88.6%) and 4 (11.4%) patients and 50 (76.9%) and 15 (23.1%) patients were CMV-positive and -negative, respectively. The difference in the CMV viral loads was not statistically significant (p=0.157) (Table 6).

**Table 6.** Distribution of CMV viral loads according to the immunosuppressive regimen.

			**CMV viral load**		**Total**
			**<800 copies**	**>800 copies**	
Regimen	**Tacrolimus based regimen**	**Count**	4	31	35
		**%**	11.4%	88.6%	
	**Cyclosporine based regimen**	**Count**	15	50	65
		**%**	23.1%	76.9%	
Total		**Count**	19	81	100
		**% of Total**	19.0%	81.0%	100.0%

CMV – Cytomegalovirus; Pearson’s Chi-Square = 2.006, p-value = 0.157.

### Distribution of CMV viral load according to diabetes and renal function status of patients

Among the 100 patients, 35 patients had diabetes, and 65 were non-diabetic. Among the diabetic patients, 31 (88.6%) and 4 (11.4%) patients were CMV-positive and -negative, respectively. On the other hand, 50 (76.9%) and 15 (23.1%) non-diabetic patients were CMV-positive and -negative, respectively. Our findings showed that the difference between the CMV load of diabetic and non-diabetic patients was not significant (p=0.157) (Table 7).

**Table 7.** Distribution of CMV viral loads according to the diabetes.

			**CMV viral load**		**Total**
			**<800 copies**	**>800 copies**	
Diabetes	**No diabetes**	**Count**	15	50	65
		**%**	23.1%	76.9%	
	**Diabetes**	**Count**	4	31	35
		**%**	11.4%	88.6%	
Total		**Count**	19	81	100
		**% of Total**	19.0%	81.0%	100.0%

CMV – Cytomegalovirus; Pearson’s Chi-Square = 2.006, p-value = 0.157.

The renal function of the patients was assessed via the serum creatinine levels. High and normal serum creatinine levels represented abnormal and normal renal function, respectively. In our study, 18 patients suffered from abnormal renal function. Among the patients with normal creatinine levels, 65 (79.3%) and 17 (20.7%) patients were CMV-positive and -negative, respectively. On the other hand, 16 (88.9%) and 2 (11.1%) patients with high creatinine levels were CMV-positive and -negative, respectively. Again, the difference between CMV loads of patients with normal and abnormal renal functions was insignificant (p=0.34) (Supplementary Table 1).

### CMV serology of recipient and donor and postoperative SVR

We conducted CMV serology for all the study participants and their respective donors prior to renal transplant. Preoperatively, a total of 53 patients were CMV-negative, out of which 10 and 43 patients received the graft from CMV-negative and -positive donors. 47 patients were CMV-positive preoperatively, of which 33 and 14 patients received the graft from CMV-negative and -positive donors. Postoperatively, five (50%), 37 (86.1%), 29 (87.9%), and 10 (71.4%) patients from the R negative D negative, R negative D positive, R positive D negative, and R positive D positive groups, respectively, were CMV-positive (Supplementary Table 2). The intergroup differences were statistically significant (p=0.03). Furthermore, as shown in Supplementary Table 3, the SVR analysis revealed all the patients to be CMV-negative at 12 weeks postoperatively.

## Discussion

We recruited a total of 100 renal transplant patients (74 males and 26 females) for this study belonging to almost all age ranges, including pediatric, young, middle-aged, and elderly adults. Our results indicated that CMV incidence did not differ significantly between males and females. However, we observed a significantly higher incidence of CMV infection among middle-aged patients (31–50 years). Our results concerning gender agreed with previous studies; however, several studies have shown no association between age and the risk of CMV infection among the post-renal transplant recipients [1, 7, 8]. Our finding could be attributed to the higher proportion of middle-aged individuals in our sample pool. We propose that including a larger study group might alter these results. We measured the ALT and serum creatinine levels to assess the renal function of the patients. Previous studies have shown low ALT levels and high serum creatinine levels associated with chronic kidney disease and abnormal renal function. It is noteworthy that the ALT and serum creatinine levels are inversely correlated [9]. Our observations corroborated these findings. However, we did not find significant differences in the proportion of patients with CMV infection in normal and high ALT or serum creatinine groups (Table 3 and Supplementary Table 1, respectively).

During solid-organ transplantation, induction and immunosuppressive therapies are used to reduce the probability of graft rejection due to the host’s immunity. We used either tacrolimus or cyclosporine as the immunosuppressive regimens. The proportions of the patients treated with either of the regimens who later developed CMV infection were not significantly different. This finding indicated that none of the immunosuppressive therapies affected the risk of CMV incidence. Our results were in accordance with those reported by Asberg *et al.*, who also showed that the use of either tacrolimus or cyclosporine did not affect the activation or treatment of CMV infection in organ transplant recipients [10]. In addition, the patients were induced with either basiliximab or ATG. However, a significantly higher proportion of ATG-induced patients were CMV-positive, which indicated that the patients induced by ATG were at higher risk of CMV infection. Our findings agreed with those reported previously in elderly kidney transplant patients [11]. On the contrary, some studies have reported no significant effect of induction therapies on CMV incidence in renal transplant patients [12]. Another study also reported no difference in the incidence of respiratory viral infection in pediatric lung transplant patients induced with either ATG or basiliximab [13].

Several studies have reported no significant correlation between preoperative diabetes and risk of CMV infection [7, 8]. Another study conducted by Beyler *et al.* also showed a limited association between preoperative diabetes and postoperative CMV incidence; however, they attributed their results to a small number of patients and a short follow-up period [14]. Still, the results of these studies agreed with those in our study. Interestingly, the association between post-transplantation CMV infection and new-onset diabetes mellitus after transplantation (NODAT) remains debatable [15–17].

Hartman A *et al.* showed that post-transplantation CMV incidence was three times higher in patients belonging to the D positive R negative group than those in D negative R positive and D positive R positive groups [18]. In their review, Requião-Moura *et al.* reported that a combination of D positive R negative and late prophylaxis leads to a high risk of developing resistance to the antiviral treatment [1]. In our study, we observed that the recipients belonging to the D positive R negative, and D negative R positive groups showed a significantly higher risk of CMV infection than the other two groups.

We used Valcyte 450 tablets to administer CMV prophylaxis in recipients who were CMV-positive postoperatively. The main active ingredient of this medicine is valganciclovir. This oral regimen has been widely used to manage CMV infection in solid organ transplant patients [19, 20]. On receiving Valcyte 450 orally, we observed that the CMV load decreased to <800 copies/mL in all the patients, which was retained till 12 weeks postoperatively.

There were a few limitations of this study. The sample size in this study was small, and a higher number of patients might have revealed the association of CMV infection with novel factors and further elaborated the predictive efficiencies of the factors identified in this study to be associated with the risk of CMV infection. In addition, this study was conducted at a single center, which might affect the generalization of our findings. None of the patients were followed up for more than 12 weeks postoperatively. Hence, we could not assess the CMV incidence in the patients after that period and the probability of the association of CMV incidence with the renal transplant procedure. Future studies must focus on increasing the sample size and recruiting the patients from multiple centers spread across a large demographic region, which might help better generalize the results and assess the effects of differences in demographic characteristics on the CMV infection risk status in post-renal transplant recipients. In addition, these studies must include a longer follow-up period to further elucidate the risk of transplant-related CMV incidence after the termination of the CMV prophylaxis.

## Conclusion

Our findings revealed that the risk of CMV infection among renal transplant patients is associated with several factors. Middle-aged individuals (30–50 years old), regardless of gender, exhibited a higher infection risk than patients of other age groups. Furthermore, ATG induction and the difference in the preoperative CMV infection status of both donor and recipient significantly affected the risk of CMV infection in post-transplant recipients. However, the type of immunosuppressive regimen did not affect the risk of CMV infection. Interestingly, the renal function of the patients and the presence of any comorbidities, such as diabetes, were not associated with CMV infection risk. We propose that future studies must be focused on a larger study sample to reduce any bias and further elucidate the relationship between CMV incidence and potential risk factors in renal transplant patients.

## Acknowledgments

### Conflict of interest

The authors declare no conflict of interest.

### Ethical approval

The study was approved by the Ethics Committee) of the College of Medicine, University of Basrah (approval number/ID: 2021-03040817, 1/3/2021).

### Consent to participate

Written informed consent was obtained from the participants.

### Personal thanks

We thank all persons who supported us during our study at the Nephrology and Dialysis Center in Basrah teaching hospitals.

### Authorship

MYAA contributed to conceptualizing, methodology, and writing the original draft. HS contributed to editing the manuscript, data collection, data curation, and data analysis.

## Supplementary Material

Supplemental data file.

**Table 1.** Distribution of CMV viral loads according to the renal function.

			**CMV viral load**		**Total**
			**<800 copies**	**>800 copies**	
**Renal function Test**	**Normal serum creatinine**	**Count**	17	65	82
		**%**	20.7%	79.3%	
	**Increased serum creatinine**	**Count**	2	16	18
		**%**	11.1%	88.9%	
**Total**		**Count**	19	81	100
		**% of Total**	19.0%	81.0%	100.0%

CMV – Cytomegalovirus; Pearson's Chi-Square = 0.88, p-value = 0.34.

**Table 2.** Distribution of CMV viral loads based on CMV serology of donor (D) and recipient (R) status prior to renal transplant.

			**CMV viral load**		**Total**
			**<800 copies**	**>800 copies**	
**Serology of donor (D) and recipient (R)**	**R negative D negative**	**Count**	5	5	10
		**%**	50.0%	50.0%	100.0%
	**R positive D positive**	**Count**	4	10	14
		**%**	28.6%	71.4%	100.0%
	**R negative** **D positive**	**Count**	6	37	43
		**%**	13.9%	86.1%	100.0%
	**R positive** **D negative**	**Count**	4	29	33
		**%**	12.1%	87.9%	100.0%
**Total**		**Count**	19	81	100
		**% of Total**	19.0%	81.0%	100.0%

CMV – Cytomegalovirus; Pearson's Chi-Square = 8.804, p-value = 0.03.

**Table 3.** Distribution of CMV viral loads according to the SVR at 12 weeks.

			**CMV viral load**		**Total**
			**<800 copies**	**>800 copies**	
**SVR**	**SVR**	**Count**	100	0	100
		**%**	100.0%	0.0%	100.0%
**Total**		**Count**	100	0	100
		**% of Total**	100.0%	0.0%	100.0%

CMV – Cytomegalovirus; SVR – Sustained Virologic Response.
