# Supplementary material for: Cytomegalovirus infection after renal transplantation
Source: J Med Life. 2022 Jan;15(1):71–7. doi: 10.25122/jml-2021-0209 (PMC8852648; doi:10.25122/jml-2021-0209)
Supplement: Supplemental data file. [file JMedLife-15-71-S.pdf]

Supplemental data file.

Table 1. Distribution of CMV viral loads according to the renal function.

			CMV viral load		Total
			<800 copies	>800 copies	
Renal function Test	Normal serum creatinine	Count	17	65	82
		%	20.7%	79.3%	
	Increased serum creatinine	Count	2	16	18
		%	11.1%	88.9%	
Total		Count	19	81	100
		% of Total	19.0%	81.0%	100.0%

CMV – Cytomegalovirus; Pearson's Chi-Square = 0.88, p-value = 0.34.

Table 2. Distribution of CMV viral loads based on CMV serology of donor (D) and recipient (R) status prior to renal transplant.

			CMV viral load		Total
			<800 copies	>800 copies	
Serology of donor (D) and recipient (R)	R negative D negative	Count	5	5	10
		%	50.0%	50.0%	100.0%
	R positive D positive	Count	4	10	14
		%	28.6%	71.4%	100.0%
	R negative D positive	Count	6	37	43
		%	13.9%	86.1%	100.0%
	R positive D negative	Count	4	29	33
		%	12.1%	87.9%	100.0%
Total		Count	19	81	100
		% of Total	19.0%	81.0%	100.0%

CMV – Cytomegalovirus; Pearson's Chi-Square = 8.804, p-value = 0.03.

Table 3. Distribution of CMV viral loads according to the SVR at 12 weeks.

			CMV viral load		Total
			<800 copies	>800 copies	
SVR	SVR	Count	100	0	100
		%	100.0%	0.0%	100.0%
Total		Count	100	0	100
		% of Total	100.0%	0.0%	100.0%

CMV – Cytomegalovirus; SVR – Sustained Virologic Response.
